# Psychological Capital Relates With Teacher Enjoyment: The Mediating Role of Reappraisal

**DOI:** 10.3389/fpsyg.2022.879312

**Published:** 2022-05-12

**Authors:** Xiang Zhou, Songyun Zheng

**Affiliations:** College of Foreign Languages, Shanghai Maritime University, Shanghai, China

**Keywords:** psychological capital, reappraisal, teaching enjoyment, emotion regulation, online teaching

## Abstract

This study examined the relationship between psychological capital (PsyCap) and teacher enjoyment in the context of online teaching and investigated whether the emotion regulation (ER) strategy of reappraisal mediated their relationship. 221 Chinese university teachers were selected as the research sample through snowball sampling in an online survey. After controlling for age, gender, teaching experience, education level, time and energy input during online teaching and online teaching experience, the results showed that PsyCap and reappraisal positively influence the teachers’ online teaching enjoyment (OTE), and reappraisal significantly mediated the relationship between teachers’ PsyCap and OTE, suggesting that optimistic and resilient teachers with more self-efficacy and hope are more likely to find enjoyment during online teaching, and high PsyCap combined with the use of reappraisal leads to greater OTE. The study not only confirms the positive role of reappraisal as an emotion regulation strategy in online teaching, but also provides practical implications for the realization of enjoyable online teaching experience.

## Introduction

Over the past few years, online teaching has gained worldwide popularity among students and teachers as a welcoming way which incorporates multimedia resources and technology to maximize learning efficiency (Stricker et al., 2011; Oza and Naik, 2016; Serrano et al., 2019). Against the backdrop of the COVID-19 pandemic outbreak back in 2020, online teaching becomes not only preferable but essential as students can learn and teachers can deliver lectures without being physically gathered in classrooms. As the pandemic keeps coming back with different variants such as Delta and Omicron, it is still necessary under various circumstances for teachers worldwide, including those in China, to teach online. What is more important, as blended learning is becoming increasingly important in an extensive range of disciplines (Holbeck and Hartman, 2018; Gonzalez and Knecht, 2020; Veerapen et al., 2020), it is necessary to switch our attention not only to learners (Holbeck and Hartman, 2018; Gillis and Krull, 2020; Okada and Sheehy, 2020; Kohnke et al., 2021; Maheshwari, 2021; Wang and Jiang, 2022), but further to teachers, whose degree of enjoyment during online teaching has an impact on both their own well-being (Anderson et al., 2021; Creely et al., 2021; Mahmood et al., 2021), and the emotion, well-being and learning enjoyment of students (Badia et al., 2019; Eloff et al., 2021; Ergun and Dewaele, 2021; Goenner, 2021; Meishar-Tal and Levenberg, 2021; Naylor and Nyanjom, 2021).

However, when facing the changes of switching the offline teaching mode to online, teachers have experienced various negative emotions such as anxiety (Gao and Zhang, 2020; Li et al., 2020), stress (Ozamiz-Etxebarria et al., 2021), depression (Santamaria et al., 2021), and burnout (Moe and Katz, 2020; Kotowski et al., 2022). Nevertheless, blended teaching and learning, as an inevitable trend initiated by the development of technology and hugely promoted worldwide partly due to the pandemic, enjoy an increasing popularity among students and teachers of almost all levels of education. Therefore, what deserves special attention but so far has been largely ignored is how teachers can accept and more preferably take a positive attitude toward online teaching. With the exception of teachers’ satisfaction (Pham et al., 2021; Fute et al., 2022; Li and Yu, 2022; Reeves et al., 2022), few studies have been devoted to teachers’ positive emotions in online settings, especially teaching enjoyment.

Previous research shows that PsyCap is helpful to stimulate an individual’s positive psychological capacities and resources (Luthans et al., 2006, 2007). Previous studies also have confirmed the significantly positive relationships between PsyCap and desirable employee attitudes such as job satisfaction, organizational commitment, and psychological well-being (Avey et al., 2011). However, few studies, if any, have been carried out thus far to investigate the relationship between PsyCap and teaching enjoyment. On the other hand, although there have been studies exploring teachers’ emotion regulation goals and strategies and their psychological impacts (Gong et al., 2013; Jiang et al., 2020), it remains unknown whether the use of particular emotion regulation strategies helps to increase teachers’ enjoyment in online teaching. This study, therefore, aims to examine whether PsyCap predicts online teaching enjoyment and whether the emotion regulation strategy of reappraisal exerts a mediating effect on the relationship between PsyCap and OTE by surveying a sample of Chinese university teachers with online teaching experience.

## Literature Review

### Psychological Capital and Emotions in Teaching

Psychological capital, a core positive construct in positive psychology, has been developed by Luthans (2002) and Luthans and Youssef (2004) and defined as an individual’s positive psychological state of development characterized by four dimensions: (1) efficacy: having confidence to take on and put in the necessary effort to succeed at challenging tasks; (2) optimism: making a positive attribution about succeeding now and in the future; (3) hope: persevering toward goals and, when necessary, redirecting paths to goals in order to succeed; (4) resilience: when beset by problems and adversity, sustaining and bouncing back and even beyond to attain success (Luthans et al., 2007:3). Previous research suggests that the effect of the combined construct PsyCap is a stronger predictor of achievement than the independent effect of its four components (Luthans et al., 2007). By promoting the experience of positive emotions and the reduction of negative emotions in workplaces, PsyCap helps people to achieve better outcomes. Avey et al. (2011) found significant positive relationships between PsyCap and positive employee attitudes, desirable employee behaviors, and performance, but negative relationships between PsyCap and negative employee attitudes and behaviors by carrying out a meta-analysis.

It is notable that research on the relationship between PsyCap and teacher emotions is still in the initial stage of development. Whereas teachers with positive attitudes and emotions are more likely to create a happy and enjoyable atmosphere in class, those with negative emotions might discourage their students, which is unfavorable to the effectiveness of teaching and learning. It has been found that PsyCap positively predicted the psychological well-being and happiness of university teachers (Li, 2018; Kun and Gadanecz, 2022), and helps them thrive in career development (Liu et al., 2021). Three of the four dimensions of PsyCap, namely optimism, hope, and resilience were also proved to have a significant impact on secondary school teachers’ job satisfaction (Mikus and Teoh, 2021). No study to date, however, has examined whether there is a relationship between teachers’ PsyCap and teaching enjoyment in the context of online teaching.

### Teachers’ Emotion Regulation and Reappraisal

Emotion regulation refers to attempts to influence which emotions individuals have, when they have them, and how they experience and express them (Gross, 1998). The process model of emotion regulation makes the prediction that different emotion regulation strategies should have different consequences for how a person feels, thinks, and acts, both immediately and over the longer term (Gross, 2015). The two most commonly explored ER strategies are reappraisal and suppression. Reappraisal is defined as a cognitive change, which is formed by reinterpreting the negative emotional stimulus that allows the involved person to stay open-minded, discover positive aspects, and find options to act. Thus, the negative emotional impact can be decreased as people shift their perspectives and start to perceive things positively (Gross, 2002; Gross and John, 2003). By contrast, suppression refers to an inhibition of the inner negative feelings by showing a positive or neutral one, which decreases behavioral expression, but fails to decrease emotion experience (Gross, 2002). Gross and John (2003) claimed that reappraisal is more useful for regulating aversive emotions, because cognitive change is associated with significantly more positive emotions, a reduction in negative emotions, a greater psychological well-being, and an increased interpersonal functioning. Previous research has revealed that reappraisal, not suppression, is beneficial to teachers’ well-being and thus to the realization of a motivating teaching style (Han et al., 2020; Moe and Katz, 2021). Furthermore, it has been demonstrated that the use of reappraisal, but not expressive suppression, was significantly correlated with lower levels of teachers’ emotional exhaustion (Donker et al., 2020). Hence close examination needs to be carried out concerning how reappraisal helps to increase teachers’ positive emotions.

On a daily basis, teachers work in a more emotionally demanding atmosphere than most other professions (Brotheridge and Grandey, 2002). Teaching effectiveness and the relationship between teachers and students can be influenced considerably by teachers’ emotions and emotion regulation in the classroom. Teachers accordingly would often try to upregulate their positive emotions and downregulate their negative emotions in the classroom by using ER strategies (Taxer and Frenzel, 2015). Research has also been conducted into the impact of teachers’ ER on such positive psychological constructs as teachers’ work engagement (Greenier et al., 2021) and self-efficacy beliefs (Uzuntiryaki-Kondakci et al., 2020), and the association of teachers’ use of ER strategies and such negative constructs as emotional exhaustion (Donker et al., 2020) and teacher burnout (Chang, 2020). Overall, the appropriate use of ER strategies, especially reappraisal, has been found positively correlated with positive psychological constructs but negatively associated with negative constructs. In view of the previous research on ER strategies and the positive role of reappraisal, this study further explores the mediating role of reappraisal in the relationship between PsyCap and OTE.

### Teaching Enjoyment

Inspired by the development of positive psychology, the study of positive emotions has been attracting constant attention from scholars and practitioners in education (Ganotice et al., 2016; Wang et al., 2021b). As the most commonly reported positive emotion, enjoyment in the field of education has been explored mainly in the form of learning enjoyment from the learners’ perspective (Jiang and Dewaele, 2019; Fang and Tang, 2021; Li et al., 2021; Zhang and Tsung, 2021). Research on teaching enjoyment, however, is still few and far between. Since emotion is contagious, teaching effectiveness and learners’ motivation are supposed to be influenced by teachers’ emotional states. Hence teaching enjoyment deserves as much, if not more, of our examination as learning enjoyment.

Previous studies mainly focus on the teaching enjoyment of teachers from a single discipline. For example, Mierzwa (2019) investigated foreign language teaching enjoyment and foreign language learning enjoyment experienced by foreign language teachers in Poland, the results of which indicated that teachers experienced a relatively high level of foreign language teaching enjoyment and foreign language learning enjoyment. More recently, Marban et al. (2021) explored the factors that have an impact on the enjoyment of teaching mathematics, the results of which showed that anxiety has a significant and strong influence on the enjoyment of mathematics teaching. Likewise, Jiang et al. (2021) found in their survey study that Chinese mathematics teachers experienced enjoyment most frequently, followed by satisfaction, anxiety, and anger. Ergun and Dewaele (2021) investigated the relationship between well-being, resilience and foreign language teaching enjoyment and found that resilience, one of the dimensions of PsyCap, was the strongest predictor of foreign language teaching enjoyment.

Overall, a review of literature on teaching enjoyment shows that little research has been conducted on teaching enjoyment of either discipline-general teachers or university teachers in an online context. It still remains unclear what makes enjoyable online teaching. Therefore, the current study aims to empirically investigate whether PsyCap predicts OTE and whether reappraisal mediated the relationship between PsyCap and OTE with a sample of Chinese university teachers.

### Research Hypotheses

As stated above, previous studies have confirmed that there is a positive association between PsyCap and positive emotions such as job satisfaction (Luthans et al., 2007) and psychological well-being (Ganotice et al., 2016; Huang and Zhang, 2021; Mikus and Teoh, 2021) and a negative correlation between PsyCap and negative emotions such as job stress (Narsa and Wijayanti, 2021) and burnout (Freire et al., 2020; Barratt and Duran, 2021; Wang et al., 2021a). However, the question remains unsettled as to whether PsyCap would lead to more enjoyable online teaching experience. Hence the present study proposed the following two research hypotheses:

Hypothesis 1: PsyCap positively predicts online teaching enjoyment.
Hypothesis 2: Reappraisal mediates the relationship between PsyCap and online teaching enjoyment.

Specifically, in an attempt to explore whether positive psychological state and positive cognitive change relate with a higher level of teacher enjoyment, it is hypothesized that PsyCap (independent variable) is positively correlated with the use of the ER strategy of reappraisal (mediator), which might play a positive and mediating role in the relationship between PsyCap and teacher enjoyment during online teaching (dependent variable).

## Materials and Methods

### Procedures

After obtaining ethical approval from the authors’ institution, 221 Chinese university teachers were invited through snowball sampling to participate in this study via WeChat, one of the dominant social networking applications in China. All the respondents participated on a voluntary basis and they were informed in the invitation letter of the purpose of the study and the confidentiality of the survey data.

### Participants

A total of 221 Chinese university teachers from different parts of China participated in the study (all valid; 100%) after receiving an invitation or finding the call for participation on WeChat groups. The majority were female (*N* = 147; 66.5%). Age was between 24 and 60 (Mean = 39.8; SD = 7.9). Their teaching experience ranged from 0.5 to 42 years (Mean = 13.4; SD = 9.2). As for education level, almost half of the participants have a Master’s degree (107; 48.4%), 98 of them (44.3%) have a Ph.D. degree, and only 16 (7.2%) participants hold a Bachelor’s degree. The participants’ online teaching experience ranged from one semester (*N* = 94; 42.5%), two semesters (*N* = 79; 35.7%), three to four semesters (*N* = 42; 19%), to five or more than five semesters (*N* = 6; 2.7%). A question was also asked as to whether and how the time and energy they put in online teaching had changed compared with the traditional offline teaching. A majority of the participants reported that it had increased (*N* = 145; 65.6%), only 18 of them (8.1%) reported a decrease in the time and energy input, and 58 of them (26.2%) claimed that it remained almost unchanged. Further demographic information is presented in Table 1.

**TABLE 1:** Demographic characteristics of the sample (*N* = 221).

	*N*	%
**Gender**		
Female	147	66.5%
Male	74	33.5%
**Age**		
20–29	14	6.3%
30–39	101	45.7%
40–49	76	34.4%
50–59	28	12.7%
60+	2	0.9%
**Teaching experience**		
<4	56	25.6%
5–10	36	11.4%
11–20	87	39.7%
21–30	29	13.2%
31+	11	5%
**Education background**		
Bachelor’s degree	16	7.2%
Master’s degree	107	48.4%
Ph.D. degree	98	44.3%
**Time and energy input: online vs. offline teaching**		
Unchanged	58	26.2%
Increased	145	65.6%
Decreased	18	8.1%

### Materials

#### Psychological Capital

The teachers’ PsyCap was measured with 12 items adapted from Luthans et al. (2007). The shortened PsyCap scale was comprised of four subscales: (1) efficacy (three items, e.g., “I feel confident analyzing a long-term problem to find a solution”); (2) hope (three items, e.g., “At the present time, I’m energetically pursuing my work goals”); (3) resilience (three items, e.g., “I can be “on my own,” so to speak, at work if I have to”); (4) optimism (three items, e.g., “When things are uncertain for me at work, I usually expect the best”). Participants were asked to rate their level of agreement on the descriptions of PsyCap on a five-point Likert scale ranging from 1 (“totally disagree”) to 5 (“totally agree”). The Cronbach’s alpha coefficient of the overall PsyCap scale (see Appendix) was 0.85, which shows good reliability.

#### Reappraisal

Reappraisal was measured with five items adapted from the model of emotion regulation in Gross and John (2003), e.g., “When I want to feel more positive emotion (such as joy or amusement), I change what I’m thinking about.” Teachers were asked to rate their use of reappraisal during online teaching on a five-point Likert scale ranging from 1 (“totally disagree”) to 5 (“totally agree”). The Cronbach’s alpha coefficient of the reappraisal scale (see Appendix) was 0.83.

#### Online Teaching Enjoyment

Teachers’ online teaching enjoyment was assessed with ten items by a scale adapted from Ergun and Dewaele (2021). We adapted the scale to the settings of online teaching and the Chinese context, e.g., “I’m confident to tackle the technological problems that come up during online teaching;” “My students and I form a tight group during online teaching and we often communicate on online platforms such as WeChat, Chaoxing, and Rain Classroom.” Participants were also asked to rate their level of agreement on the descriptions of OTE on a five-point Likert scale ranging from 1 (“totally disagree”) to 5 (“totally agree”). The Cronbach’s alpha coefficient of the OTE scale (see Appendix) was 0.85, indicating a good reliability.

#### Control Variables

Age, gender, teaching experience, education level, time and energy input during online teaching compared with offline teaching, and online teaching experience were chosen in the current study as control variables to counteract the potential confounding effects (Luthans and Youssef-Morgan, 2017).

### Data Analysis

SPSS 22.0 was adopted to conduct data analysis in the present study. After using Harman’s single-factor test to assess the common method bias, we made correlation analysis of the relationships between PsyCap, reappraisal, and online teaching enjoyment. Then SPSS macro PROCESS v3.5 (Hayes, 2017) was adopted to examine the mediating effect of reappraisal on the relationship between PsyCap and OTE.

## Results

### Assessment of Common Method Biases

In this study, Harman’s single-factor test was used to assess the common method bias. It was found that a single factor accounted for 22.5% of the variance, which is below the critical value of 40% (Podsakoff et al., 2003), indicating that there is no serious common method bias in the current study.

### Descriptive Statistics and Correlation Analysis

The results of the descriptive statistics and correlation analysis of PsyCap, reappraisal, and OTE are displayed in Table 2. As expected, PsyCap is positively correlated with both reappraisal (γ = 0.53, *p* < 0.01) and OTE (γ = 0.36, *p* < 0.01). Likewise, reappraisal is positively associated with OTE (γ = 0.41, *p* < 0.01).

**TABLE 2:** Descriptive statistics and correlation analysis (*N* = 221).

	M (SD)	1	2	3
(1) PsyCap	3.76 (0.05)	1		
(2) Reappraisal	3.75 (0.61)	0.53**	1	
(3) OTE	3.45 (0.62)	0.36**	0.41**	1

**p < 0.01.

### Mediation Analysis

**FIGURE 1:**
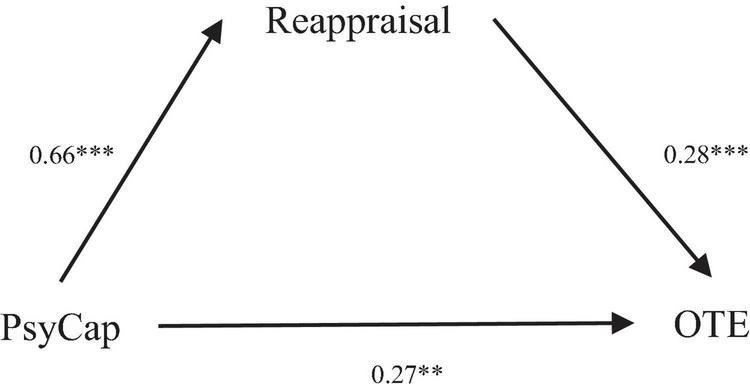
The mediating model of Psychological Capital (PsyCap), reappraisal, and online teaching enjoyment. ***p < 0.001; **p < 0.01.

SPSS macro PROCESS was used in this study to examine the relationships among PsyCap, reappraisal, and OTE and the mediating role of reappraisal between PsyCap and OTE among Chinese university teachers. Mediation model testing was conducted to estimate the parameters of two regression equations: (1) the direct effect of PsyCap on OTE; (2) the indirect effect (mediation effect) of reappraisal on the relationship between PsyCap and OTE. The results of regression analysis are displayed in Table 3. The results show that PsyCap positively predicts both OTE (β = 0.46, *t* = 5.71, *p* < 0.001) and reappraisal (β = 0.66, *t* = 9.15, *p* < 0.001), and reappraisal positively predicts OTE (β = 0.28, *t* = 3.83, *p* < 0.001). As shown in the mediation model in Figure 1, reappraisal plays a mediating role in the relationship between PsyCap and OTE. Specifically, the relationship was partially mediated by reappraisal as the effect of PsyCap on OTE was reduced by 0.19 from (β = 0.46, *p* < 0.001) to (β = 0.27, *p* < 0.01).

**TABLE 3:** Test of mediation effect.

Variable	Model 1			Model 2		

	OTE			OTE		

	Effect of value	SE	*t*	Effect of value	SE	*t*
PsyCap	0.46	0.08	5.71***	0.27	0.09	2.95**
Reappaisal				0.28	0.07	3.83***
R^2^		0.21			0.15	
*F*		7.95***			6.41***	

**p < 0.01, ***p < 0.001.

To further test the reliability of the mediating effect, non-parametric bootstrapping method with 5,000 times of resampling was used. As shown in Table 4, the value of the direct effect of PsyCap on OTE is 0.27, accounting for 59% of the total effect, with a 95% confidence interval (CI) of [0.09, 0.45]. The indirect effect of reappraisal between PsyCap and OTE is 0.19, accounting for 41% of the total effect. The 95% CI is [0.05, 0.32], which indicates that there is a significant mediating effect of reappraisal on the relationship between PsyCap and OTE.

**TABLE 4:** Bootstrap analysis of significance test of mediation effect.

Path	Effect of value	Effect of amount	Bootstrap 95% CIs
Direct effect	0.27	59%	[0.09. 0.45]
PsyCap → OTE			
Indirect effect	0.19	41%	[0.05, 0.32]
PsyCap → Reappraisal → OTE			
Total effect	0.46	100%	[0.29, 0.62]

## Discussion

### Main Findings

First of all, the results reveal a positive relationship between PsyCap and online teaching enjoyment, which confirms the first hypothesis in this study and is consistent with previous research on the connection between school PsyCap and achievement emotions (Ergun and Dewaele, 2021; Kang and Wu, 2021). Previous studies have found the positive correlation between school PsyCap and positive emotions such as enjoyment, hope, and pride among particular teaching communities (Ergun and Dewaele, 2021; Kang and Wu, 2021). Similarly, the findings in this study have confirmed the positive correlation between PsyCap and teaching enjoyment among teachers of all disciplines in the online settings. It indicates that optimistic and resilient teachers with more self-efficacy and hope are more likely to find enjoyment during online teaching. As shown in Table 1, 145 participants (65.6%) reported that they had put significantly more time and energy in online teaching than traditional classroom teaching, and more of them (*N* = 183; 82.8%) reported that their salary remained unchanged during the period of online teaching, showing that online classroom is a more demanding and stressful job context. Furthermore, it is more difficult for teachers to establish effective and adequate communication with their students and colleagues in the online context, all of which calls more strongly for teachers’ positive emotions. Teachers with high PsyCap are more likely to think in a positive and optimistic way and realize that online teaching not only has distinct advantages but also is the irreversible trend in an increasingly digitalized era. Therefore, based on the survey results, it is expected that teachers engaged with online teaching will be encouraged and trained in teacher training programs to have higher PsyCap, including its four dimensions of efficacy, hope, resilience, and optimism, for PsyCap positively predicts OTE and OTE is beneficial to online teaching and learning.

The study also reveals an important mediating role of reappraisal as an ER strategy in connecting PsyCap with online teaching enjoyment, which confirms the second hypothesis and is a new finding in this field of study. This indicates that teachers with a high level of PsyCap are more likely to reappraise a negative or stressful situation, to reconsider it from a positive perspective, and teachers who use more of the ER strategy of reappraisal tend to have greater enjoyment in online teaching. The findings, in line with previous research on the positive role of reappraisal (Chang, 2020; Donker et al., 2020; Han et al., 2020), are reflective of the importance of reappraisal which helps to improve university teachers’ enjoyment and well-being and facilitates positive cognitive changes during online teaching. To be more specific, the use of reappraisal enables university teachers to experience more enjoyment during online teaching. Furthermore, positive psychological resources combined with the use of reappraisal enable teachers to find more enjoyment and probably experience less burnout in online teaching, which motivate them to guarantee and constantly improve their online teaching quality.

### Theoretical Contribution

Online teaching is enjoying more and more popularity and is playing an increasingly important role among teachers and students worldwide. Hence there is an urgent need at the present time to explore what can help boost online teaching enjoyment and if the use of some emotion regulation strategies can help teachers obtain an enjoyable rather than anxiety-dominated experience when delivering classes online or when organizing online activities for a blended course.

Based on Ergun and Dewaele (2021), a novel construct Online Teaching Enjoyment was proposed and investigated, which provides new insights into the factors that can affect teachers’ enjoyment in an online teaching context. By revealing both the direct effect of PsyCap on OTE and the indirect effect of cognitive reappraisal on the relationship between PsyCap and OTE, this research study contributes to the fields of both teacher psychology and positive psychology.

### Practical Implications

The results provided practical implications for finding an effective approach to improve university teachers’ positive emotions in the context of online teaching. As it is necessary during the pandemic and recommendable in the development of a hybrid course to accommodate diversified resources into one course for teachers to engage with online teaching, it is vitally important to seek out ways allowing university teachers to keep a positive emotional state when preparing and delivering their online lectures. To help teachers find more enjoyment in online teaching, colleges and universities should try to strengthen their psychological capital and train them to make better use of the ER strategy of reappraisal, making them more optimistic, confident and resilient. As university teachers face various pressures such as publication and teaching (Boyd and Harris, 2010; Tian and Lu, 2017), this study provides a timely implication in a period that emphasizes more than ever the necessity of online teaching for university faculty to embrace the strategy of reappraisal in order to be more resilient and benefit from a more positive state of mind. Only in this way can they leverage the advantages of technology to improve their teaching efficiency, and undergo less burnout and enjoy more positive feelings whenever it is necessary to switch the face-to-face teaching mode or the blended learning mode to a completely online version or whenever they would like to deliver blended teaching. Therefore, it is suggested that more teacher training programs, online, offline, or combined, should be organized to help teachers raise more PsyCap, encourage them to use ER strategies more effectively, and become better aware of the advantages of online teaching so as to make full use of it.

### Limitations and Further Avenues

As one of the initial attempts to explore the relationship between PsyCap, reappraisal, and OTE, this study promoted empirical understanding of their relationship in the context of online teaching, contributing to the literature of teacher psychology. Despite the significance of the study, there are still some limitations. First, the research design was cross-sectional and causal inference cannot be made. Second, the data were collected by using questionnaire survey, which might contain biases. Future studies might adopt a longitudinal or mixed-method research design. Third, this research was restricted to reappraisal as the mediator. Future research might choose more constructs as mediators between PsyCap and OTE. Finally, this survey study used only Chinese teachers as the sample. Future studies might adopt an intercultural perspective by inviting teachers from different countries to participate in the research.

## Data Availability Statement

The raw data supporting the conclusions of this article will be made available by the authors, without undue reservation.

## Ethics Statement

The studies involving human participants were reviewed and approved by Department of Foreign Languages, Shanghai Maritime University. The patients/participants provided their written informed consent to participate in this study.

## Author Contributions

XZ: research design, data collection, data analysis, and the writing and editing of the article. SZ: research design, data collection, drawing tables and figures, and the writing and editing of the article. Both authors contributed to the article and approved the submitted version.

## Conflict of Interest

The authors declare that the research was conducted in the absence of any commercial or financial relationships that could be construed as a potential conflict of interest.

## Publisher’s Note

All claims expressed in this article are solely those of the authors and do not necessarily represent those of their affiliated organizations, or those of the publisher, the editors and the reviewers. Any product that may be evaluated in this article, or claim that may be made by its manufacturer, is not guaranteed or endorsed by the publisher.

## Appendix

### *Psychological Capital Scale* (Chinese Version Was Used)

(1) I feel confident analyzing a long-term problem to find a solution.
(2) I don’t feel confident helping to set targets/goals in my work area (R).
(3) I feel confident contacting people outside the institution to discuss problems.
(4) At the present time, I’m energetically pursuing my work goals.
(5) I cannot think of many ways to reach my current work goals (R).
(6) At this time, I am meeting the work goals that I have set for myself.
(7) I usually manage difficulties one way or another at work.
(8) I can be “on my own,” so to speak, at work if I have to.
(9) I usually feel difficult to deal with stressful things at work.
(10) When things are uncertain for me at work, I usually expect the best.
(11) I’m optimistic about what will happen to me in the future as it pertains to work.
(12) I approach this job as if “every cloud has a silver lining.”

### *Reappraisal Scale* (Chinese Version Was Used)

(1) When I want to feel more positive (such as joy or amusement), I change what I’m thinking about.
(2) When I want to feel less negative emotion, I change the way I’m thinking about the situation.
(3) When I want to feel more positive emotion, I change the way I’m thinking about the situation.
(4) When I want to feel less negative emotion (such as sadness or anger), I change what I’m thinking about.
(5) When I’m faced with a stressful situation, I make myself think about it in a way that helps me calm down.

### *Online Teaching Enjoyment Scale* (Chinese Version Was Used)

(1) The students are friendly during online teaching.
(2) The students are not very supportive during online teaching (R).
(3) The colleagues help each other during online teaching.
(4) I feel proud of my progress and accomplishments during online teaching.
(5) I feel confident to tackle the technological problems that come up in online teaching.
(6) I don’t like online teaching (R).
(7) It’s fun to do online teaching.
(8) My students and I form a tight group during online teaching and we often communicate on online platforms such as WeChat, Chaoxing, and Rain Classroom.
(9) I feel my students appreciate the time and energy I have put in online teaching.
(10) The students don’t like my online class (R).
